# An assessment of cord ferritin concentration and its predictors among a cohort of Canadian preterm and term infants

**DOI:** 10.1017/S0007114524003362

**Published:** 2025-02-14

**Authors:** Lulu X. Pei, Jennifer A. Hutcheon, Crystal D. Karakochuk

**Affiliations:** 1 Food, Nutrition and Health, The University of British Columbia, Vancouver, Canada; 2 Healthy Starts, BC Children’s Hospital Research Institute, Vancouver, Canada; 3 Obstetrics and Gynaecology, The University of British Columbia, Vancouver, Canada

**Keywords:** Cord blood, Infant Fe status, Fe deficiency, Pregnancy, Ferritin

## Abstract

Low iron (Fe) stores at birth may adversely influence child cognitive and motor development. The aims of this study were to assess cord blood Fe levels and explore maternal and neonatal factors associated with Fe status. Cord blood specimens (*n* 46) were obtained from the BC Children’s Hospital BioBank in Vancouver, Canada. The primary outcome was cord plasma ferritin, measured using sandwich-ELISA. Predictors of interest included maternal age, gestational age, gravidity, infant sex, birth weight and delivery method. Median (interquartile range (IQR)) maternal age and gestational age at delivery was 33·5 (29·3–35·8) years and 36·5 (30·0–39·0) weeks, respectively, and 44 % of infants were female. Median (IQR) cord ferritin was 100·4 (75·7–128·9) µg/l, and 26 % had low Fe status (ferritin <76 µg/l). Among preterm deliveries, a 1-week increase in gestational age was associated with a 6·22 (95 % CI (1·10, 9·52)) µg/l increase in median cord ferritin. However, among term deliveries, a negative trend was observed (–2·38 µg/l per week of gestation (95 % CI (–34·8, 0·78))), indicating a potential non-linear relationship between gestational age and cord ferritin. Female term infants had higher cord ferritin compared with males (*β* (95 % CI): 30·3 (18·4, 57·9) µg/l), suggesting sex-specific differences in Fe transfer, acquisition and utilisation. Cord ferritin was higher with vaginal deliveries compared with caesarean sections (*β* (95 % CI): 39·1 (29·0, 51·5) µg/l). Low Fe status may be a concern among infants in Canada; however, further research is needed to inform appropriate thresholds to define optimal Fe status in cord blood.

Iron (Fe) is an essential micronutrient, playing an important role in various cellular functions, including oxidative energy metabolism, erythropoiesis and transport of oxygen to body tissues^(1)^. Fe is vital for early brain growth and function because of its role in neurogenesis, brain development and myelination^(2)^. As such, Fe deficiency in fetal or neonatal life may adversely influence cognitive and motor development, with long-term consequences that are difficult to remedy, persisting into adulthood^(2)^. Understanding the relationship between maternal and fetal Fe status may help to diagnose and prevent Fe deficiency in pregnancy and infancy and therefore improve health outcomes.

We recently reported a high prevalence of Fe deficiency among a cohort of sixty pregnant individuals in Vancouver, Canada, with over 80 % having probable Fe deficiency (ferritin <30 µg/l) in late pregnancy (24–38 weeks gestation)^(3)^. This raised concerns over whether infants are also Fe-deficient. However, given the challenges of blood specimen collection in newborns and infants, literature on the Fe status of this population is limited. Cord ferritin concentration is a commonly used indicator of fetal Fe status, reflective of fetal tissue Fe stores. Previous studies have demonstrated a significant positive association between maternal and cord ferritin concentrations^(4–6)^, with the effect being more pronounced when maternal Fe status is compromised^(7–9)^. Cord ferritin has also been shown to be a strong predictor of Fe status during the first 2 years of life^(10,11)^, highlighting the clinical importance of low Fe stores at birth and later risk of Fe deficiency. Tamura *et al.* further reported poorer mental and psychomotor development, including language ability, fine-motor skills and tractability, at 5 years of age among children in the lowest cord ferritin quartile (<76 µg/l) among a cohort of 278 children from Alabama, USA^(4)^. This emphasises a window of opportunity during the prenatal period to optimise development.

This study summarises Fe and inflammation status of cord blood specimens from a cohort of infants born in Vancouver, Canada. Additional exploratory analyses are presented to examine maternal and neonatal factors associated with Fe status that may serve as priority area targets for future research.

## Methods

### Study population

Our study population was drawn from deliveries occurring between August 2016 and August 2022 at BC Women’s Hospital in Vancouver, Canada. Pregnant individuals consented to provide umbilical cord blood specimens to the BC Children’s Hospital BioBank (BCCHB) for future use for research purposes. Ethical approval was obtained from the University of British Columbia Children’s and Women’s Research Ethics Board (H22-03587). A total of forty-six specimens were obtained from twenty-three term and twenty-three preterm infants. This included all available term specimens at the BCCHB and an equal number of randomly selected preterm specimens to facilitate statistical comparisons between delivery types.

### Laboratory measurements

Umbilical cord blood specimens were collected in vacutainer tubes containing EDTA, following clinical protocols. Specimens were centrifuged at 1500 x *g* for 10 min at room temperature within 3 h of specimen collection, and plasma aliquots were stored at –80°C until analysis. Cord plasma was assessed using sandwich-ELISA to measure ferritin (µg/l), soluble transferrin receptor (sTfR; mg/l), α1-acid glycoprotein (AGP; g/l) and C-reactive protein (CRP; mg/l)^(12)^.

### Iron status

The primary outcome for this analysis was cord plasma ferritin concentration (µg/l), an indicator of fetal tissue Fe stores^(6)^. Low Fe status was defined as a cord plasma ferritin concentration <76 µg/l^(4,7)^, and severe Fe deficiency was defined as cord ferritin <34 µg/l, a level indicative of suspected brain Fe deficiency^(13)^. We also assessed cord plasma sTfR concentration (mg/l) and two biomarkers of inflammation: AGP (g/l) and CRP (mg/l), reflective of acute and chronic inflammation, respectively.

### Statistical analysis

Characteristics of the study cohort and measures of Fe status were summarised using medians with interquartile ranges (IQR). Predictors of interest included gestational age at delivery, maternal age, gravidity, infant sex, birth weight and delivery method (vaginal or caesarean). Univariable quantile regression was used to quantify the association between each predictor and Fe status separately, while multivariable quantile regression was used to quantify the association independent of other predictors, computed by the rank inversion method^(14)^.

Analyses were conducted for the overall cohort as well as stratified by gestational age at delivery into preterm (gestational age <37 weeks) and term (gestational age ≥37 weeks) deliveries, which represents a natural clinical dichotomy of subgroups with differential risk for Fe deficiency^(15)^. In the case of non-linear patterns, sensitivity analysis was conducted by visualising data using a non-parametric locally estimated scatterplot smoothing (LOESS) approach.

## Results

Fe status assessment was performed on forty-six cord blood specimens, consisting of twenty-three preterm and twenty-three term specimens. Table 1 summarises birth and delivery characteristics. Median (IQR) maternal age and gestational age at delivery were 33·5 (29·3–35·8) years and 36·5 (30·0–39·0) weeks, respectively. The majority of deliveries were through caesarean section (89 %, *n* 39). Median (IQR) birth weight was 2570 (1316–3320) g, and 44 % (*n* 20) of infants were female.

**Table 1.** Summary of birth and delivery characteristics*

	All (*n* 46)		Delivery type			
	Median or *n*	Interquartile range or %	Preterm (*n* 23)		Term (*n* 23)	
			Median or *n*	Interquartile range or %	Median or *n*	Interquartile range or %
Maternal age (years)	33·5	29·3–35·8	33·0	28·5–37·0	34·0	31·5–35·0
Gravidity	2·0	1·0–3·0	2·0	1·0–3·5	2·0	1·5–3·0
Delivery method						
Vaginal delivery	5	11·4	4	18·2	1	4·6
Caesarean section	39	88·6	18	81·8	21	95·5
Gestational age (weeks)	36·5	30·0–39·0	30·0	28·5–32·0	39·0	38·5–39·5
Birth weight (g)	2570	1316–3320	1294	1096–1765	3385	2952–3650
Infant sex						
Male	25	55·6	12	54·5	13	56·5
Female	20	44·4	10	45·5	10	43·5

* Values are median (interquartile range) or *n* (%).

Table 2 summarises biomarkers of Fe status and inflammation. Inflammation was low in the cord plasma specimens based on accepted thresholds of acute and chronic indicators of inflammation; the majority (*n* 44) had undetectable levels of CRP (<0·2 mg/l) and forty-five had AGP < 1 g/l. Because of the very low levels of inflammation, ferritin concentrations were not adjusted for inflammation. Median (IQR) cord plasma ferritin concentration was 100·4 (75·7–128·9) µg/l. Overall, 26 % of infants (*n* 12) had low Fe status, based on cord plasma ferritin <76 µg/l, with a greater proportion in the preterm cohort (39 %, *n* 9 *v*. 13 %, *n* 3 in the term cohort). Only one infant had severe Fe deficiency, based on cord plasma ferritin <34 µg/l.

**Table 2.** Summary of biomarkers of iron status and inflammation in cord plasma*

	All (*n* 46)		Delivery type			
	Median or *n*	Interquartile range or %	Preterm (*n* 23)		Term (*n* 23)	
			Median or *n*	Interquartile range or %	Median or *n*	Interquartile range or %
Inflammation biomarkers						
CRP < 0·2 mg/l	44	95·7	21	91·3	23	100
AGP (g/l)	0·09	0·07–0·13	0·08	0·06–0·11	0·12	0·09–0·18
Fe biomarkers						
Ferritin (µg/l)	100·4	75·7–128·9	86·2	50·3–124·9	109·9	89·3–148·4
<34 µg/l	1	2·2	1	4·3	0	0
<76 µg/l	12	26·1	9	39·1	3	13·0
sTfR (mg/l)	6·7	5·1–7·8	6·8	5·8–7·8	5·9	4·3–7·8
>8·3 mg/l	8	17·4	5	21·7	3	13·0

CRP, C-reactive protein; AGP, α1-acid glycoprotein; sTfR, soluble transferrin receptor.

* Values are median (interquartile range) or *n* (%).

### Unadjusted associations between predictors and cord ferritin

**Figure 1.**
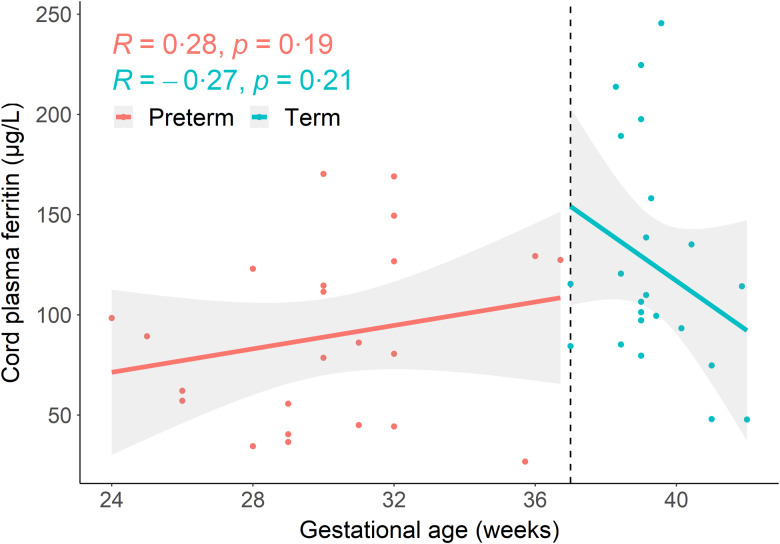
Scatterplot depicting association between gestational age at delivery and cord plasma ferritin (µg/l) by preterm or term delivery. R: Spearman’s rank correlation coefficient; lines represent fitted linear regression lines with 95 % CI shaded in grey.

The univariable analysis between gestational age and cord plasma ferritin showed a significant positive association, where a 1-week increase in gestational age was associated with a 2·81 (95 % CI (0·39, 5·29)) µg/l increase in median cord ferritin. Subgroup analysis by gestational age at delivery (preterm *v*. term) revealed a positive trend between gestational age and cord ferritin among preterm deliveries, whereas a negative trend between gestational age and cord ferritin among term deliveries (Fig. 1). Birth weight was also found to be positively associated with cord plasma ferritin (*β* (95 % CI): 7·65 (2·64, 18·1) µg/l per 500 g). This pattern was most pronounced among the preterm cohort (*β* (95 % CI): 23·9 (2·98, 54·0) µg/l per 500 g), with no significant association found among the term cohort.

**Figure 2.**
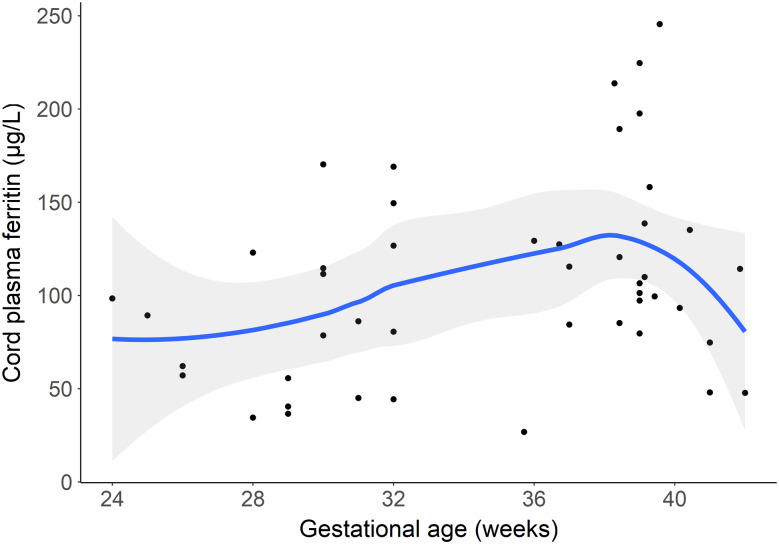
Scatterplot of gestational age at delivery and cord ferritin with fitted LOESS curve; 95 % CI shaded in grey. LOESS, locally estimated scatterplot smoothing.

Sensitivity analysis using a LOESS non-parametric approach to explore the association between the gestational age continuum and cord plasma ferritin revealed a similar non-linear pattern, where an inflection point is observed at about 38 weeks gestation (Fig. 2).

**Figure 3.**
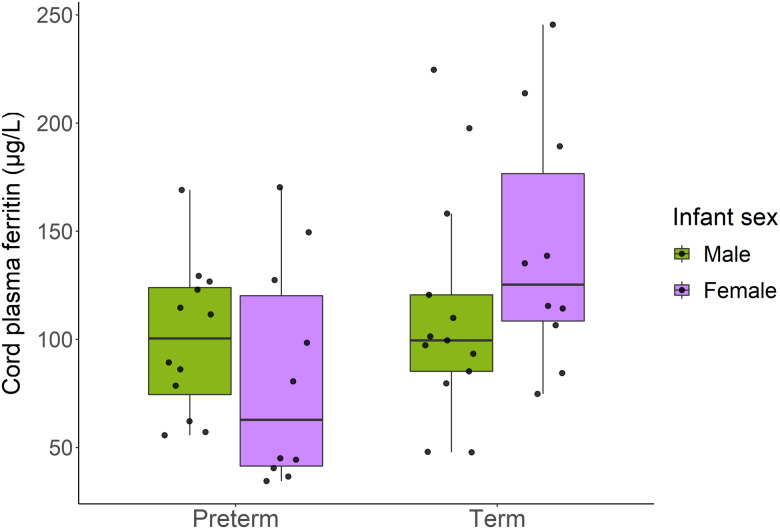
Side-by-side boxplot depicting cord plasma ferritin concentration (µg/l) by preterm *v*. term delivery and infant sex.

Univariable analysis between infant sex and cord plasma ferritin showed overall no significant difference between males and females. Subgroup analysis showed no differences among preterm infants; however, female term infants had on average a median cord ferritin 16·0 (95 % CI (3·67, 49·9)) µg/l higher than male term infants (Fig. 3).

Overall, maternal age was not significantly associated with cord plasma ferritin. However, among preterm deliveries, a positive association was found where a 1-year increase in maternal age was associated with a 3·70 (95 % CI (1·34, 6·56)) µg/l increase in median cord ferritin. Comparatively, among term deliveries, a negative association was found, where a 1-year increase in maternal age was associated with a 8·14 (95 % CI (–9·08, –0·82)) µg/l decrease in median cord ferritin. No association was observed between gravidity and cord plasma ferritin. In terms of delivery method, cord plasma ferritin was higher for vaginal deliveries compared with caesarean section deliveries (*β* (95 % CI): 24·6 (6·45, 39·0) µg/l). Subgroup analysis was not conducted due to low representation of vaginal deliveries in the term cohort.

### Adjusted associations between predictors and cord ferritin

The independent predictors in our multivariable regression models included maternal age, gravidity, gestational age, delivery method and infant sex. As expected, birth weight and gestational age were highly correlated (*r* 0·92); therefore, of the two variables, only gestational age was included in the regression models (Table 3). A 1-week increase in gestational age at delivery was associated with a 2·60 (95 % CI (0·44, 5·30)) µg/l increase in median cord plasma ferritin concentration. This positive association was most pronounced among preterm deliveries, with a 1-week increase in gestational age at delivery associated with a 6·22 (95 % CI (1·10, 9·52)) µg/l increase in median cord ferritin concentration. However, similar to the univariate analysis, a negative trend was observed among the term cohort where increasing gestational age was associated with decreasing cord ferritin.

**Table 3.** Multivariable quantile regression output modelling the outcome of cord plasma ferritin concentration (µg/l), in the overall cohort and by delivery type (preterm *v*. term)*

	All (*n* 46)		Delivery type			
	*β*	95 % CI	Preterm (*n* 23)		Term (*n* 23)	
			*β*	95 % CI	*β*	95 % CI
Maternal age (years)	1·70	–5·98, 5·01	1·14	–0·49, 4·88	–8·50	–11·8, –1·41
Gravidity	4·59	–5·74, 21·3	–3·88	–4·24, 5·50	16·8	–7·02, 21·1
Gestational age (weeks)	2·60	0·44, 5·30	6·22	1·10, 9·52	–2·38	–34·8, 0·78
Delivery method: vaginal†	39·1	29·0, 51·5	21·3	15·2, 44·1	–	
Infant sex: female‡	1·33	–30·0, 30·2	–26·4	–43·4, 19·6	30·3	18·4, 57·9

* Values are estimated difference in cord plasma ferritin concentration (95 % CI).

† *v*. reference: caesarean section. Delivery method not included in term cohort multivariable regression model due to low representation of vaginal deliveries.

‡ *v*. reference: male.

Increasing maternal age was associated with decreased cord ferritin concentration among term deliveries, but not preterm deliveries. Additionally, consistent with the univariable analysis, female term infants had higher cord ferritin concentration compared with male term infants; this sex difference was not detected among preterm deliveries. In adjusted analyses, cord plasma ferritin was also found to be higher in vaginal deliveries compared with caesarean section deliveries.

## Discussion

This paper summarises the distribution of cord blood biomarkers of Fe status and inflammation among a cohort of infants delivered in Vancouver, Canada. Inflammation was low, with the majority of specimens having undetectable CRP concentrations (below the detection limit of 0·2 mg/l). Cord plasma ferritin concentrations had high variability but were generally found to be lower than levels previously reported in literature^(16)^. Over a quarter of our study cohort had low Fe stores based on ferritin <76 µg/l. This threshold level has been associated with lower mental and psychomotor test scores in children at 5 years of age^(4)^. Given the link between low Fe status early in life and subsequent cognitive and motor development, the large proportion of infants deemed to have low Fe status gives reason for concern. Additionally, there is currently no global consensus on the appropriate thresholds to diagnose Fe deficiency using ferritin concentration in cord blood specimens. Further research with larger studies is needed to obtain consensus regarding ferritin concentration thresholds in cord blood for the diagnosis of Fe deficiency to inform downstream treatment strategies.

Consistent with previous literature^(17)^, cord ferritin concentrations in our study cohort increased with gestational age with preterm infants having overall lower cord ferritin compared with term infants. Interestingly, increasing gestational age at term was associated with decreased cord ferritin concentration. This suggests a non-linear relationship between gestational age and fetal acquisition of Fe, potentially indicating a point in time where placental transfer of Fe from mother to fetus slows and is unable to match the rate of fetal uptake of Fe, thereby resulting in a net loss of Fe in the umbilical cord blood with further increasing gestational age. Previous studies often use a single model to cover the entire range of gestational ages; our findings suggest that it may be more appropriate to model preterm and term deliveries separately, or use a non-linear approach, as there may be differing trends in the relationship between gestational age and Fe status indicators. Our results, however, are limited by a small sample size, and additional research is needed to further investigate this non-linear association and the underlying physiological mechanism(s). Decreases in ferritin concentration may also reflect a redistribution of Fe rather than low Fe status, with preferential use of Fe for erythropoietic demands at the expense of tissue and storage Fe^(18)^. In this exploratory analysis, we did not have Hb concentration available to investigate utilisation of the total body Fe pool for circulating Hb rather than ferritin storage. Future research investigating neonatal Fe sufficiency should measure cord ferritin in conjunction with other hematological indices such as Hb concentration.

Cord ferritin concentration in our study cohort was found to be comparable between male and female preterm infants; however, among term infants, females had higher cord ferritin compared with males. This indicates potential sex differences in the acquisition of Fe and specifically transfer of Fe to the fetus following 37 weeks gestation. Tamura *et al.* measured cord serum ferritin concentrations in 255 infants and found significantly higher mean ferritin concentration in female infants (166 ± 110 µg/l) compared with male infants (123 ± 77 µg/l), with a significant correlation between maternal ferritin and cord ferritin for male infants but not female infants^(19)^. More recently, Larsson *et al.* also reported a similar pattern of higher ferritin concentration among female infants compared with male infants, starting at birth (umbilical cord blood) and persisting up to 12 months of age^(20)^. In a Canadian context, recent findings from the Alberta Pregnancy Outcomes and Nutrition (APrON) cohort study demonstrated that third trimester maternal Fe status (serum ferritin, hepcidin and hepcidin:erythropoietin) was significantly higher in pregnant individuals carrying male infants compared with those carrying female infants^(21)^. Interpretation of this finding is limited as cord blood specimens were not obtained; however, this result may suggest that there is less mobilisation of maternal Fe to male fetuses by this gestational age, consistent with our finding that male term infants had lower cord plasma ferritin concentration compared with female term infants. The exact mechanism for these differences is largely unknown; however, taken together, these findings suggest the need for further investigation of the mechanistic underpinnings of these sex differences as well as development of sex-specific recommendations and guidelines for assessing infant Fe status.

Additionally, we found that maternal age was negatively associated with cord ferritin concentration among term infants, but not preterm infants. This may be a result of increased risk of adverse health conditions associated with advancing maternal age, such as hypertensive complications, predisposing a pregnant individual to a greater risk of Fe deficiency and therefore impaired transfer of Fe to the fetus. However, we were not able to discern this from our data due to a small sample size and incomplete data on maternal health and pregnancy complications. The presence of this association only among term infants is consistent with the previously proposed hypothesis, whereby a point of exhaustion of maternal Fe stores is reached after 37 weeks gestation and placental transfer of Fe to the fetus is reduced; however, further research is warranted to explore this potential explanation.

Despite the low representation of vaginal deliveries in this cohort, cord plasma ferritin was found to be higher in vaginal deliveries compared with caesarean section deliveries. This finding is consistent with previous literature from a cohort of 120 pregnant women from Bangkok, Thailand, which found a comparable magnitude of effect (*β* (95 % CI): 32·96 (3·68, 62·24) µg/l)^(22)^. A similar pattern was also observed in the Cork BASELINE Birth Cohort Study, where among 413 cord blood specimens with ferritin measured, infants delivered vaginally had higher cord ferritin than those delivered by caesarean section (median (IQR): 193·1 (136·1, 394·8) *v*. 162·6 (111·2, 334·5) µg/l, *P* = 0·005)^(23)^. A systematic review and meta-analysis found that delivery by caesarean section, compared with vaginal delivery, was associated with a decreased level of Fe-related hematological indices, including hematocrit, Hb, and erythrocyte in cord and peripheral blood in term neonates^(24)^. This reduction in placental transfusion may be attributable to weaker transfusion force (related to factors such as uterine contraction, maternal blood pressure, delayed onset of respiration and gravity) and a shorter transfusion period associated with caesarean section deliveries^(24)^. Details of timing and methods of cord clamping were not available for this study.

Limitations of this study include the small sample size. We were restricted by the number of cord blood specimens available at our hospital’s biobank, which were limited due to requirements for deliveries to occur within the limited range of collection hours. Due to these factors, the majority of specimens obtained were from planned caesarean sections; thus, we had a low representation of vaginal deliveries. As such, the nature of this analysis was exploratory and hypothesis-generating to inform the design of future studies. We also did not have robust data regarding pregnancy complications and birth outcomes such as gestational diabetes and hypertension, fetal growth restriction, which are factors that could affect Fe metabolism. Additionally, information on maternal Fe status was not available; therefore, we could not investigate the association between maternal ferritin concentration and cord blood Fe biomarkers. Future research should aim to explore the link between markers of maternal Fe status, placental Fe content, umbilical cord ferritin concentration and breast milk Fe content to further understand the complex transfer of Fe from mother to fetus and infant and to inform Fe recommendations for pregnancy.

### Conclusion

We found evidence of a non-linear relationship between gestational age and cord ferritin concentration, highlighting the need for gestational age-specific ferritin cut-offs. Sex-specific differences were detected, where female term infants had higher cord ferritin concentration compared with male term infants. Additionally, infants delivered vaginally had higher cord ferritin concentration compared with infants delivered by caesarean section. Low Fe status may be a concern among infants in Canada; however, further research is needed to inform appropriate thresholds to define both inflammation and Fe deficiency in cord blood specimens. The estimates of cord ferritin concentrations among preterm and term infants in our study cohort can be used to inform the design of future studies seeking to investigate interventional or associative effects on this outcome.
